# Up-Regulation of Non-Homologous End-Joining by MUC1

**DOI:** 10.3390/genes15060808

**Published:** 2024-06-19

**Authors:** Tadayoshi Bessho

**Affiliations:** The Eppley Institute for Research in Cancer and Allied Diseases, Fred & Pamela Buffett Cancer Center, University of Nebraska Medical Center, 986805 Nebraska Medical Center, Omaha, NE 68198, USA; tbessho@unmc.edu; Tel.: +1-(402)559-7018

**Keywords:** double-strand break repair, MUC1, pancreatic cancer, HDAC inhibitors

## Abstract

Ionizing radiation (IR) and chemotherapy with DNA-damaging drugs such as cisplatin are vital cancer treatment options. These treatments induce double-strand breaks (DSBs) as cytotoxic DNA damage; thus, the DSB repair activity in each cancer cell significantly influences the efficacy of the treatments. Pancreatic cancers are known to be resistant to these treatments, and the overexpression of MUC1, a member of the glycoprotein mucins, is associated with IR- and chemo-resistance. Therefore, we investigated the impact of MUC1 on DSB repair. This report examined the effect of the overexpression of MUC1 on homologous recombination (HR) and non-homologous end-joining (NHEJ) using cell-based DSB repair assays. In addition, the therapeutic potential of NHEJ inhibitors including HDAC inhibitors was also studied using pancreatic cancer cell lines. The MUC1-overexpression enhances NHEJ, while partially suppressing HR. Also, MUC1-overexpressed cancer cell lines are preferentially killed by a DNA-PK inhibitor and HDAC1/2 inhibitors. Altogether, MUC1 induces metabolic changes that create an imbalance between NHEJ and HR activities, and this imbalance can be a target for selective killing by HDAC inhibitors. This is a novel mechanism of MUC1-mediated IR-resistance and will form the basis for targeting MUC1-overexpressed pancreatic cancer.

## 1. Introduction

Pancreatic cancer is one of the deadliest cancers due to difficulty detecting the disease at an early stage, resistance to conventional cancer chemotherapy, frequent recurrence, and metastasis. Recent studies have revealed that mucins could serve as potential biomarkers for early detection and as therapeutic targets in pancreatic cancers [1]. Mucins are heterogeneous high molecular-weight glycoproteins with heavy O-linked and N-linked oligosaccharides. There are 21 members of the mucin family that are either secreted or membrane-bound. Pancreatic cancer cells are characterized by aberrant expression, altered glycosylation patterns, and altered localization of mucins [2]. Those changes in mucins are believed to play crucial roles in the development and progression of various biological characteristics of pancreatic cancers.

MUC1 is a member of the glycoprotein mucins. MUC1 is co-translationally processed into two polypeptides, and then the two polypeptides form the mature transmembrane protein. MUC1 is normally expressed on the luminal surfaces of ductal epithelia, and its extensive O-glycosylation of the extracellular domain protects from various pathogens. MUC1 molecules found on cancer cells are often underglycosylated, and this underglycosylation promotes interactions of MUC1 with many transmembrane receptors and components of the extracellular matrix [3]. MUC1 consists of the extracellular domain, the transmembrane region, and the cytoplasmic tail (CT). The extracellular domain is non-covalently linked to the transmembrane region and undergoes auto-cleavage to release CT in response to various stimuli. Thus, the extracellular domain of MUC1 serves as a ligand to transduce outside signals to a 72-amino acid CT that mediates oncogenic signals by serving as a transcriptional cofactor through protein-protein interactions in the nucleus [3,4]. Furthermore, recent studies revealed MUC1 as a crucial metabolic regulator [4]. MUC1 interacts with hypoxia-inducible factor 1 α (HIF1a) and p53 through the CT, the two key transcriptional factors to control metabolic gene expressions [5,6]. MUC1 induces transcriptional alterations in metabolic reprogramming in cancer cells via the interaction with HIF1a and p53. MUC1 also regulates the expression of genes involved in metabolic pathways and modulates the metabolic flux in glycolysis, the pentose phosphate pathway, the tricarboxylic acid cycle, and fatty acid biosynthesis [5,6,7]. Thus, the overexpression of MUC1 in cancers increases the production of biosynthesis intermediates that are required for cell growth. Recent studies have also discovered interesting links between metabolic enzymes/metabolites and DSB repair [8,9]. Fumarase is a metabolic enzyme that converts malate to fumarate in the TCA cycle. Nuclear fumarase is phosphorylated after IR by DNA-PK, and the activated fumarase increases the local concentration of fumarate after IR. The locally accumulated fumarate inhibits histone demethylase KDM2, resulting in the elevated dimethylation of histone H3K36 that enhances the recruitment of KU70 to stimulate NHEJ [8]. It has also been shown that fumarate and succinate both suppress HR. Cancer-prone hereditary leiomyomatosis and renal cell cancer (HLRCC) and succinate dehydrogenase-related hereditary paraganglioma and pheochromocytoma (SDH PGL/PCC) have germline loss-of-function mutations in genes encoding the Krebs cycle enzymes fumarate hydratase and succinate dehydrogenase [9]. The cells from these syndromes have elevated levels of fumarate and succinate and display HR-deficient phenotypes that partly explain the cancer-prone phenotypes. Therefore, MUC1-induced changes in metabolites could influence the DSB repair activities and consequently the therapeutic resistance of MUC1-expressed pancreatic cancer.

One of the characteristics of pancreatic cancer is the resistance to chemotherapy. Resistance to various therapeutics, including 5-fluorouracil, cisplatin, cyclophosphamide, and gemcitabine, was found to be associated with the overexpression of MUC1 in various tissue cultures, xenograft, and clinical settings [10]. The anti-apoptotic activity of MUC1 and the suppression of intracellular uptakes of drugs by MUC1 contribute to the chemo-resistance. Interestingly, overexpression of MUC1 also makes cells resistant to ionizing radiation (IR). IR and 5-fluorouracil, cisplatin, cyclophosphamide, and gemcitabine induce double-strand breaks (DSBs) or DNA damage that is repaired by HR [11,12,13]. Curiously, the impact of DSB repair on the MUC1-mediated chemo- and radiation-resistance has not been thoroughly explored. DSBs are repaired through two major repair pathways, homologous recombination (HR) and non-homologous end-joining (NHEJ) in humans [14,15,16,17]. The molecular mechanism of DSB repair has been studied, and we now have good knowledge of the basic mechanisms of HR and NHEJ [18,19,20]. Both HR and NHEJ consist of multi-step enzymatic processes containing many DNA repair factors. ATP is an essential co-factor in both HR and NHEJ. For example, the recombinase RAD51 is an ATPase, ATP hydrolysis is required for nucleoprotein filament formation in HR, and DNA ligase IV uses ATP to ligate DNA ends to complete the NHEJ. DNA polymerases play pivotal roles in HR and NHEJ. DNA polymerase δ is required for DNA repair synthesis, extension of hybrid DNA, capture of the 5′ end of the broken end, and junction migration during HR [15,21,22,23,24]. DNA polymerase λ and μ are required for a gap-filling reaction before the ligation step in NHEJ [25,26,27]. Emerging evidence also suggests that microhomology-mediated NHEJ (MMEJ) which utilizes microhomologies to repair DSBs contributes to the repair of DSBs and cell viability in the absence of HR [28,29,30,31,32]. MMEJ is mediated by two DNA polymerases θ and δ [33,34]. Because each enzyme has an optimal substrate concentration, changes in cellular concentrations of ATP, ribonucleoside triphosphates (rNTPs), and deoxyribonucleoside triphosphates (dNTPs) by the overexpression of MUC1 could have an impact on the efficiency of DSB repair.

The hypothesis is that MUC1-induced metabolic alterations influence DSB repair activities. In this report, the impact of the MUC1-induced elevation of the dNTP pool on DSB repair was investigated using MUC1-overexpressed and -suppressed cancer cell line models. I also examined if the inhibition of the MUC1-altered DSB repair could sensitize MUC1-overexpressed pancreatic cancer cell lines. The results show that (1) MUC1-overexpression enhances NHEJ while partially suppressing HR, and (2) a DNA-PK inhibitor and HDAC inhibitors preferentially kill MUC1-overexpressed pancreatic cancer cell lines. Thus, MUC1 generates an imbalance between HR and NHEJ and, consequently, invokes IR-resistance and genetic instability. The suppression of HR by MUC1 also provokes an addiction to NHEJ for cell viability; thus, inhibition of NHEJ selectively kills MUC1-overexpressed pancreatic cancers.

## 2. Results

### 2.1. Modulation of DSB Repair by MUC1-Overexpression

**Figure 1:**
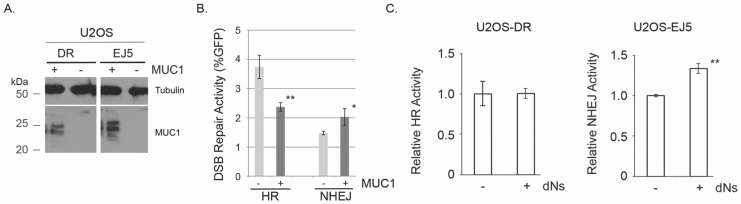
MUC1 stimulates non-homologous end-joining (NHEJ), while partially suppressing homologous recombination (HR). (**A**) Western blotting analysis of the expression of MUC1 in U2OS-DR and U2OS-EJ5. MUC1 was stably expressed in U2OS-DR cells and U2OS-EJ5 cells, which specifically detect homologous recombination (HR) and non-homologous end-joining (NHEJ), respectively. Tubulin was used as a loading control. (**B**) MUC1-overexpression stimulates NHEJ and partially suppresses HR. Cells were transfected by a plasmid with I-SceI to introduce DSBs. Successful repair of DSBs by HR or NHEJ results in the expression of GPF. DSB repair activity was expressed as %GPF positive cells by FACS. Gray and black columns indicate the DSB repair activity without and with the MUC1-overexpression, respectively. Bars indicate standard deviations obtained from at least three independent experiments. Student’s *t*-tests showed ** *p* < 0.01 and * *p* < 0.05. (**C**) The addition of deoxyribonucleosides (dNs) in the medium stimulates NHEJ but not HR. After pre-incubation of DR or EJ5 cells in the presence of 50 µM dNs for 16 h, DSB repair activities were measured by the cell-based DSB repair assay. At least three independent experiments were performed. Bars represent standard deviations. * *p* < 0.05, and ** *p* < 0.01 were obtained by Student’s *t*-test.

To examine the impact of the overexpression of MUC1 on DSB repair in human cells, we took advantage of cell-based DSB repair assays [35,36,37]. The assays utilize a GFP-reporter cassette that is integrated into a chromosome. GFP is expressed only after DSBs introduced by the I-SceI restriction enzyme are repaired. The DSB repair activity can be measured by sorting GFP-positive cells by FACS. U2OS-derived cell lines, U2OS-EJ5 and U2OS-DR, were used to specifically measure NHEJ and HR, respectively. We discovered that MUC1-overexpression increased NHEJ by ~40% (Figure 1A). Similar observations were made recently using various pancreatic cancer cell lines; however, the statuses of the expression levels of MUC1 in these cell lines have not been determined [38]. Surprisingly, HR was partially suppressed by MUC1-overexpression (Figure 1A). One possibility for the suppression of HR by MUC1-overexpression is that MUC1, directly or indirectly, suppresses the expression of critical HR factors and/or stimulates the degradation of HR factors. Cell lysates were prepared from U2OS-EJ5, with or without MUC1-overexpression, and the expression levels of several DSB repair factors including BRCA1 were examined (Supplemental Figure S1A). The expression of BRCA1 was suppressed to some extent in U2OS-EJ5 with MUC1-overexpression. RT-PCR experiments showed that this MUC1-mediated suppression of BRCA1 was caused at the transcriptional level (Supplemental Figure S1B). The data suggest that HR suppression by MUC1-overexpression is likely due to the suppression of the expression of BRCA1. Furthermore, the MUC1-overexpressed cells displayed sensitivity to two RDA52 inhibitors confirming the HR deficiency in these cells [39,40]. Altogether, MUC1 induces an imbalance in DSB repair, the stimulation of NHEJ, and the suppression of HR.

### 2.2. MUC1-under Expressed Cells Acquire an Increased Level of NHEJ by Exogenous Addition of Deoxyribonucleosides in the Cell Culture Medium

MUC1-overexpression increases dNTP pools by modulating glucose metabolism and carbon flux [7]. To investigate the impact of an increased level of the dNTP pools on NHEJ, deoxyribonucleosides (dNs) were added to the cell culture medium. The exogenous addition of dNs is known to increase the cellular dNTP pools. MUC1-underexpressed U2OS-EJ5 were grown in the medium supplemented with 50 µM dNs, and the NHEJ activity was examined. The addition of dNs in the medium stimulated NHEJ in U2OS-EJ5 (Figure 1B). It is also noted that the addition of dNs to the medium did not alter the HR activity measured by U2OS-DR; thus, elevated dNTPs do not have an impact on HR (Figure 1B). These results strongly indicate that an increased dNTP pool stimulates NHEJ.

### 2.3. Selective Killing of MUC1-Overexpressed Cell Lines by DNA-PK Inhibitor and HDAC Inhibitors 

**Figure 2:**
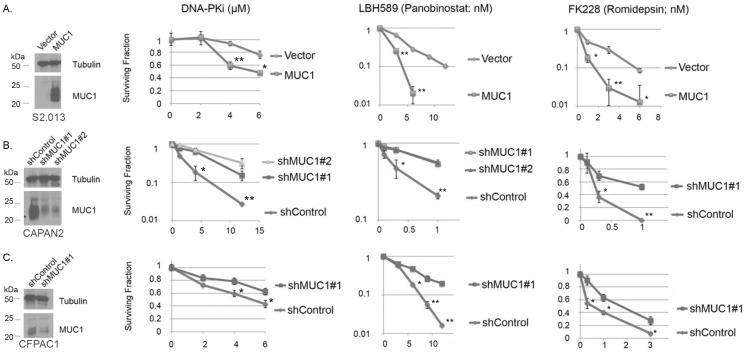
Pharmacological inhibitions of NHEJ preferentially kill MUC1-expressed pancreatic cancer cell lines. The expression of MUC1 in each cell line was confirmed by Western blotting, shown on the left. (**A**) S2.013 with and without MUC1 overexpression, (**B**) CAPAN2 with and without suppression of endogenous MUC1 by shRNA, and (**C**) CFPAC1 with and without suppression of endogenous MUC1 by shRNA. The MUC1-expressed cells showed a higher sensitivity to NU7441 (DNA-PK inhibitor), HDAC inhibitor LBH589 (pan-HDAC inhibitor), and FK228 (selective HDAC1 and 2 inhibitor), compared to the cells with low/little MUC1 expression. The cytotoxicity of each chemical was examined by the colony-forming assay. Cells were treated with indicated concentrations of chemicals until colonies formed. The error bars represent standard deviations from three independent experiments. * *p* < 0.05 and ** *p* < 0.01 determined by Student’s *t*-test.

The figure shows that MUC1 partially suppresses HR and enhances NHEJ. This imbalance in DSB repair might create the NHEJ-dependency for cell viability. Many cancer cells experience DNA replicative stress (RS), and HR is a major pathway to overcome RS [41,42,43,44]. Therefore, MUC1-overexpressed cells with a low HR activity and a high NHEJ activity may rely on NHEJ for their viability. To examine the impact of the inhibition of NHEJ on the viability of the MUC1-over expressed cancer cells, a DNA-PK inhibitor, NU7441 was used. Because HDAC1 and HDAC2 are reported to stimulate NHEJ [45], HDAC inhibitors, LBH589 (pan-HDAC inhibitor) and FK228 (HDAC1 and HDAC2 inhibitor) were also tested. The expression of MUC1 was manipulated by overexpression in S2.013 and by suppression by shRNAs in CAPAN2 and CFPAC1 (shown in Figure 2A–C). MUC1-expressed cells (S2.013 MUC1, CAPAN2, and CFPAC1) were killed preferentially by NU7441, LBH589, and FK228 compared to the cells that express lower levels of MUC1 (S2.013.Neo, CAPAN2 with shMUC1, and CFPAC1 with shMUC1). A less effective HDAC inhibitor, MS275, that inhibits HDAC1 and HDAC3 also showed a similar preferential killing of MUC1-overexpressed pancreatic cancers (Supplemental Figure S2). HDAC1 is the enzyme that is inhibited commonly by LBH589, FK228, and MS275; thus, MUC1-overexpressed pancreatic cancer cells are selectively killed by the inhibition of HDAC1.

## 3. Discussion

Pancreatic cancer has the lowest survival rate, and the current therapeutic options for advanced pancreatic cancer include radiation therapy and chemotherapy. Unfortunately, these therapeutic options do not improve the survival rate due to cellular resistance to the therapy. This report presented the results that MUC1 stimulates NHEJ while suppressing HR and this MUC1-induced imbalance in DSB repair activity sensitizes pancreatic cancer cell lines to the inhibition of NHEJ.

How the increased level of dNTP pools stimulates NHEJ is still enigmatic. To investigate the impact of MUC1 on the NHEJ activity in pancreatic cancer cell lines, the IR-induced recruitment of one of the NHEJ factors XRCC4 to chromatin was investigated (Supplemental Figure S3). XRCC4 was recruited faster and more efficiently after IR in the MUC1-overexpressed pancreatic cancer cell line (S2.013+MUC1) than MUC1-underexpressed counterparts (S2.013.Neo). The XRCC4-recruitment peaked at 2 h after IR in the MUC1-overexpressed S2.013 and at 4 h in S2.013.Neo. About 50% more XRCC4 was recruited in the MUC1-overexpressed S2.013 compared to S2.013.Neo. These results suggest that MUC1 stimulates NHEJ by enhancing the recruitment of NHEJ factors to damaged chromatin in pancreatic cancer cell line S2.013. Furthermore, an in vitro NHEJ assay with Xenopus egg extracts (Supplemental Figure S4A) [46] was performed to investigate the direct impact of the concentrations of dNTPs on the NHEJ activity. By increasing the concentrations of dNTPs, the NHEJ activity was enhanced in vitro (Supplemental Figure S4B). Significantly, a high concentration of dNTPs induces small deletions that were not detected with standard reaction conditions (Supplemental Figure S4C). These data indicate that increased concentrations of dNTPs directly stimulate mutagenic NHEJ. The enhanced NHEJ activity by MUC1 could contribute to the resistance to chemotherapeutics that induce DSBs and also to mutagenesis to initiate and/or accelerate pancreatic cancer formation. Future studies are required to decipher the mechanism of the MUC1-induced stimulation of NHEJ and the impact of MUC1-induced imbalance in the DSB repair pathways on the mutagenesis in pancreatic cancer.

The results in Figure 1A demonstrated that MUC1 overexpression partially suppresses HR. The increased NHEJ activity often leads to a reduction of HR (and vice versa) [47]. However, because the increased concentrations of dNTPs enhance NHEJ without altering HR (Figure 1C), the reduced HR in MUC1 overexpressed cells is not due to the enhanced NHEJ. The preliminary data also showed the MUC1-induced suppression of the BRCA1 expression at the transcriptional level (Supplemental Figure S1B). Rajabi et al. recently reported that MUC1 activates EZH2 histone methyltransferase and suppresses the expression of BRCA1 [48]. They showed that the EZH2 inhibitor GSK343 reactivated the expression of BRCA1 in breast cancer cell lines. However, the expression of BRCA1 in MUC1-overexpressed U2OS-EJ5 cells was not altered by GSK343 (Supplemental Figure S1C). The data showed that, in pancreatic cancers, the MUC1-EZH2 axis is not the major contributor to the suppression of BRCA1. Interestingly, hypoxia suppresses HR, and the suppression of several HR factors, including BRCA1, RAD51, and RAD52 is believed to be attributed to hypoxia-induced HR suppression [49,50,51,52]. HIF1a is a major hypoxia-induced transcription factor that regulates many genes [53,54]. Because MUC1 stabilizes HIF1a at the protein level [5,6], MUC1 could suppress BRCA1 through HIF1a and suppress HR in pancreatic cancer cells, although it has not been examined if HIF1a regulates these HR genes in non-hypoxic conditions. It will be interesting to investigate the contribution of HIF1a in the regulation of DSB repair pathways by MUC1.

MUC1-induced imbalance in DSB repair with suppressed HR and enhanced NHEJ generates a unique opportunity for selective killing of MUC1-overexpressed pancreatic cancers by RAD52 inhibitors (Supplemental Figure S1D–F) and HDAC inhibitors, LBH589 and FK228 (Figure 2). Inhibition of RAD52 is known to be synthetic lethal with an HR deficiency including BRCA1 and BRCA2 mutated breast and ovarian cancers. The MUC1-induced partial suppression of BRCA1 is sufficient to cause the synthetic lethality with RAD52 inhibitors, indicating a potential benefit of the RAD52 inhibitors to the treatment of MUC1-expressed pancreatic cancers. Significantly, PARP inhibitor Olaparib did not sensitize the pancreatic cancer cell line S2.013 with the MUC1-overexpression (Supplemental Figure S1D), supporting the observation that RAD52 inhibitors can be effective in PARP inhibitor-resistant cancers [39]. HDAC1 and HDAC2 are implicated in promoting NHEJ [45], and the selective killing of MUC1-overexpressed pancreatic cancer cells by FK228 that selectively inhibits HDAC1 and HDAC2 supports the idea that MUC1-induced imbalance in DSB repair creates a NHEJ-dependency in cell viability of MUC1-overexpressed pancreatic cancer cells.

## 4. Conclusions

MUC1 is a critical metabolic regulator in pancreatic cancer cells. The results presented in this report demonstrated that MUC1 up-regulates the NHEJ pathway while suppressing HR and this MUC1-induced imbalance in the DSB repair activity makes MUC1-overexpressed pancreatic cancers vulnerable to the pharmacological inhibition of NHEJ. Thus, the selective inhibition of NHEJ is a potential therapeutic to MUC1-overexpressed pancreatic cancers. Future studies using orthotopic implantation models and PDX (Patient-derived xenograft) models will evaluate the efficacy and selectivity of the NHEJ inhibition therapy.

## 5. Materials and Methods

### 5.1. Cell Culture

Pancreatic cancer cell line S2.013 with MUC1 overexpression (S2.013 MUC1) and MUC1-knockdown cell lines CAPAN2-shMUC1 and CFPAC1-shMUC1 [5,7,46] are a generous gift from Dr. Pankaj Singh (OU Health Stephenson Cancer Center, University of Oklahoma Health Sciences Center). MUC1-overexpressed U2OS cell lines to measure DSB repair activities were generated by transfecting the pcDNA3 harboring the MUC1 gene [47] and G418-resistant cells were selected.

### 5.2. Cell-Based DSB Repair Assay

Non-homologous end-joining (NHEJ) activity and homologous recombination (HR) activity were measured with the cell-based DSB repair assays [35]. U2OS-EJ5 and U2OS-DR were used to measure NHEJ and HR activity, respectively (generous gifts from Dr. Jeremy Stark, Beckman Research Institute of the City of Hope). U2OS-EJ5 was seeded at 10^5^ cells per well, and U2OS-DR was seeded at 5 × 10^4^ cells per well in a 12-well plate one day before transfection. The expression plasmid of I-SceI endonuclease (a generous gift from Dr. Maria Jasin at Memorial Sloan Kettering Cancer Center) was transfected to introduce DSBs. Each DSB repair activity was determined by FACS three days after transfection [39]. U2OS-EJ5 and U2OS-DR were grown and maintained in DMEM high glucose (Cytiba SH30022.01) supplemented with 10% Fetal Bovine Serum (FBS, Gibco A5256701)

### 5.3. Chemical Reagents

DNA-PK inhibitor NU7441 (Catalog# S2638) and HDAC inhibitors LBH589 (Catalog#S1030) and FK228 (Catalog#S3020) were purchased from Selleck Chemicals (Houston, TX, USA). Four deoxynucleosides, deoxyadenosine (Catalog#D7400), deoxyguanosine (Catalog#D7145), deoxycytidine (Catalog#D3897), and thymidine (Catalog#T9250), were purchased from Millipore Sigma (St. Louis, MO, USA).

### 5.4. Western Blotting

Equal amounts of cell lysates were separated by SDS-PAGE. The separated proteins were transferred to PVDF membranes. After blocking in 5% milk in TBST, the membranes were incubated with primary antibodies overnight at 4 °C with rocking. After extensive washing with TBST, the membranes were incubated with secondary antibodies at room temperature for two hours. After extensive washing with TBST, the membranes were treated with ECL reagents (BioRad, Clarity Western ECL substrate, Catalog#1705060, Hercules, CA, USA) and then exposed on X-ray film (Fuji Film, Tokyo, Japan). Primary antibodies against MUC1-CT (Armenian Hamster monoclonal antibody, a generous gift from Dr. Pankaj Singh), BRCA1 (Bethyl Laboratory Catalog#A300-000A), and β-tubulin (Cell Signaling Technology Catalog#2128, Danvers, MA, USA), and secondary antibody conjugated with horse radish peroxidase (Cytiva, Catalog#NA931 and NA934, Shinjuku, Tokyo, Japan) were used.

### 5.5. Clonogenic Survival Assay

Clonogenic assays were performed to determine cellular sensitivity to NHEJ inhibitors. Cells were seeded in 24-well plates and grown overnight. The plating efficiency of each cell line was (1) 20–25% for S2.013 and 15–20% for S2.013.MUC1, (2) 5–7.5% for CAPAN2 and 5–10% for CAPAN2 with shMUC1, and (3) 7.5–10% for CFPAC1 and 7.5–10% for CFPAC1 with shMUC1. The cells were treated with the indicated concentrations of the inhibitors until colonies formed. The colonies formed were washed with PBS gently, fixed in methanol, stained with 0.4% crystal violet (Sigma-Aldrich, Catalog# C6158, St. Louis, MO, USA) in 25% methanol, and manually counted. The surviving fraction at each concentration was determined as a ratio of the number of colonies in the treated wells to those in the non-treated wells.

## Data Availability

The original contributions presented in the study are included in the article/Supplementary Material, further inquiries can be directed to the corresponding author.

## Supplementary Materials

The following supporting information can be downloaded at: https://www.mdpi.com/article/10.3390/genes15060808/s1, Supplemental Figure S1: MUC1 suppresses the expression of BRCA1 and sensitizes the cells to RAD52 inhibitors. Supplemental Figure S2: HDAC1/3 inhibitor MS275 preferentially killed MUC1-expressing pancreatic cancer cell lines. Supplemental Figure S3: MUC1 promotes the recruitment of XRCC4 to chromatin after IR. Supplemental Figure S4: Increased dNTPs concentrations stimulate in vitro NHEJ activity. Ref. [55] is cited in Supplementary Materials.
