# Supplementary material for: Up-Regulation of Non-Homologous End-Joining by MUC1
Source: Genes (Basel). 2024 Jun 19;15(6):808. doi: 10.3390/genes15060808 (PMC11203369; doi:10.3390/genes15060808)
Supplement: Supplementary file 1 [file genes-15-00808-s001.zip › genes-3024787-supplementary.pdf]

**Supplemental Information**

Supplemental Figure S1: MUC1 suppresses the expression of BRCA1 and sensitizes the cells to RAD52 inhibitors.

Supplemental Figure S2. HDAC1/3 inhibitor MS275 preferentially killed the MUC1-expressed pancreatic cancer cell lines.

Supplemental Figure S3: MUC1 promotes the recruitment of XRCC4 to chromatin after IR.

Supplemental Figure S4: Increased dNTPs concentrations stimulate in vitro NHEJ activity.

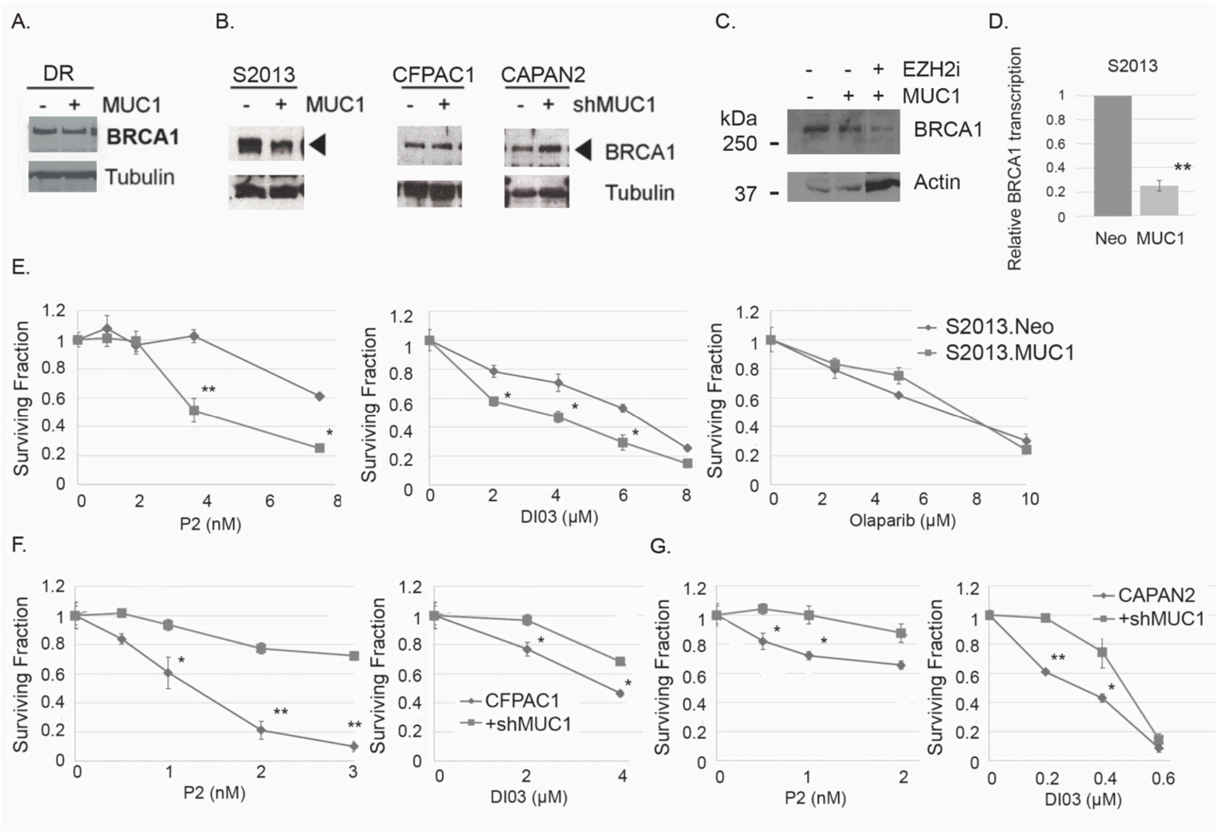


**Supplemental Figure S1.** MUC1 suppresses the expression of BRCA1 and sensitizes the cells to RAD52 inhibitors. (A) MUC1 suppresses the expression of BRCA1 in U2OS-DR. Wester blotting showed about a 70% reduction of BRCA1 in the MUC1-overexpressed U2OS-DR compared to the control U2OS-DR. No significant changes were observed in the expression of KU80 (one of the NHEJ factors) and RPA. Tubulin was used as a loading control. (B) An inverse relationship between MUC1 and BRCA1 in pancreatic cancer cell lines. BRCA1 was decreased at ~50% by MUC1 overexpression in S2.013. The suppression of MUC1 in CFPAC1 and CAPAN2 increased the BRCA1 expression by ~1.5-fold and ~4-fold, respectively. (C) Inhibition of EZH2 does not restore the expression of BRCA1 in MUC1-overexpressed U2OS EJ5. The EJ5 cells were treated with EZH2 inhibitor GSK343 at 20 μM for three days and the expression of BRCA1 was examined. The MUC1-suppressed BRCA1 was not restored by GSK343. Actin was used as a loading control. (D) MUC1 negatively regulates the transcription of the BRCA1 gene. Total mRNA was purified from S2.013.Neo and S2.013.MUC1 and RT-PCR was performed. The GAPDH gene was used as a control. PCR primers, 5'-CCTCTACTGTCCTGGCTACTA-3' and 5'-CAGATTTCCAAGGGAGACTTCA-3' were used for the BRCA1 gene, and 5'-CCATGGAGAAGGCTGGGG-3' and 5'-CAAAGTTGTCATGGATGACC-3' were used for the tubulin expression. The BRCA1 expression was normalized by the GAPDH expression. Relative BRCA1 expression level in S2013.MUC1 to one in S2013.Neo was determined. Three independent experiments were performed. Bars represent standard deviations. Student t-test showed $^{**}p<0.01$. (E-G) RAD52 inhibitors preferentially killed the MUC1-expressed pancreatic cancer cell lines. Cytotoxicity of chemicals was examined by clonogenic assay. Cells, S2.013±MUC1, CFPAC1±shMUC1, and CAPAN2±shMUC1, were incubated with the indicated concentrations of two RAD52 inhibitors, mitoxantrone (P2) [39] and DI03 [40], until colonies formed. The error bars represent standard deviations from three independent experiments. $^{*}p<0.05$ and $^{**}p<0.01$ determined by Student t-test.

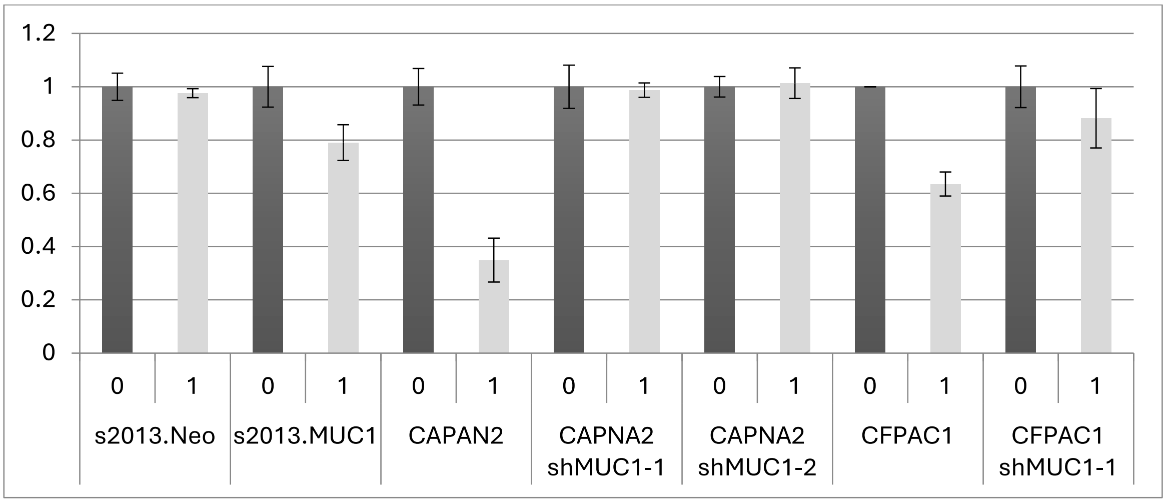


**Supplemental Figure S2.** HDAC1/3 inhibitor MS275 preferentially killed the MUC1-expressed pancreatic cancer cell lines. Cytotoxicity of MS275 was examined by clonogenic assay. Cells, S2.013±MUC1, CFPAC1±shMUC1, and CAPAN2±shMUC1, were incubated with 1 μM MS275 until colonies formed. The error bars represent standard deviations from three independent experiments.

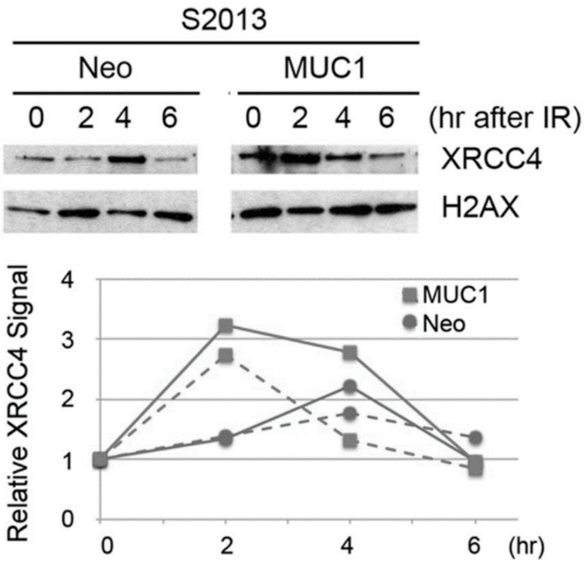


**Supplemental Figure S3.** MUC1 promotes the recruitment of XRCC4 to chromatin after IR. Cells were irradiated with IR at 10 Gy and were harvested at the indicated time. Chromatin fractions were prepared by the method described by Ward and Chen [55]. The presence of XRCC4 was examined by western blots. Histone H2AX was used as a loading control. Relative signals of XRCC4 to time "0" were plotted after the XRCC4 signals were normalized by the H2AX signals at each time point. The peak of the XRCC4 recruitment was 2 hr and 4 hr after IR in S2.013.MUC1 and S2.013.Neo, respectively. Solid lines: experiment #1 and Dot lines: experiment #2.

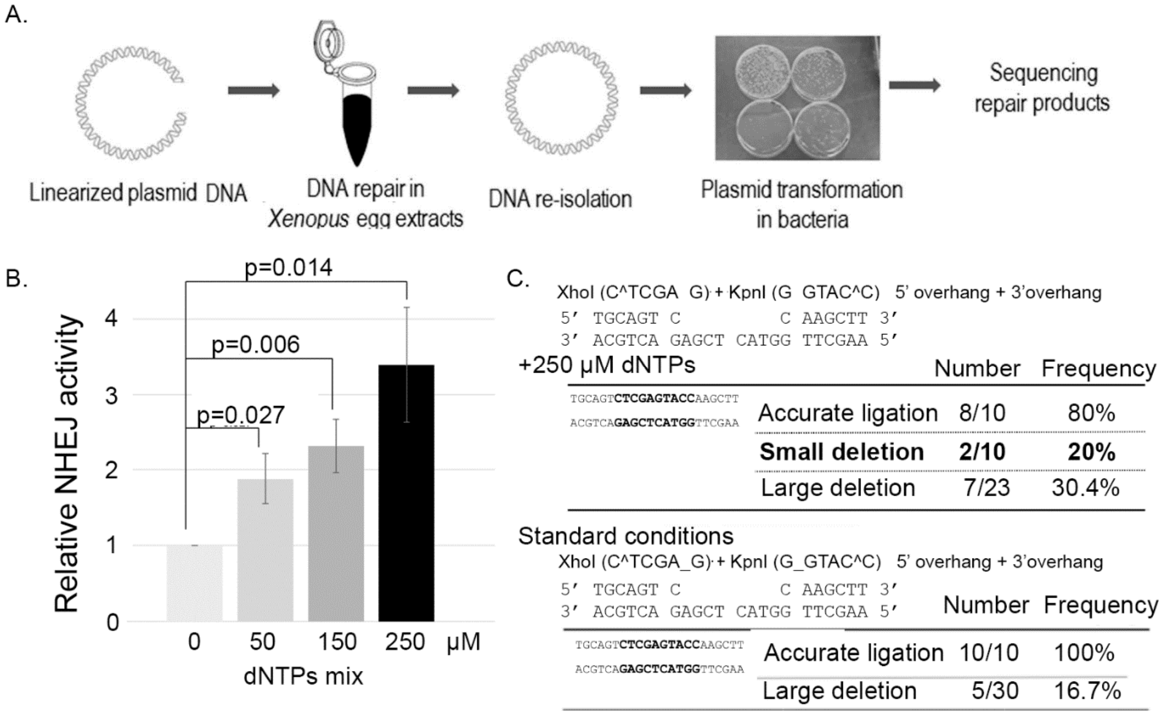


**Supplemental Figure S4.** Increased dNTPs concentrations stimulate in vitro NHEJ activity. (A) In vitro NEHJ assay with Xenopus egg extracts [46]. Briefly, Plasmid DNA was linearized by restriction endonucleases (Xho1 and Kpn1) and incubated in *Xenopus* egg extracts. The plasmid DNA was then re-isolated from extracts and transformed into bacteria cells. The repair of the DNA template would result in the formation of bacteria colonies, which can be quantified to measure the efficiency of DNA repair. Each colony contains a single clone of the repair product that can be subjected to sequencing analysis. (B) Increased concentrations of dNTPs stimulate the NHEJ activity in vitro. In the standard conditions, no dNTPs were added to the cell extract. (C) Increased dNTPs shift to error-prone NHEJ pathways. The repaired products were sequenced after the in vitro reaction. With 250 µM dNTPs, the repaired products contained small deletions (10-20 in length: 20%) while no such product was detected with the reaction under the standard conditions. Large deletions (0.2 -2 kbp) were detected by analyzing repaired plasmid sizes with agarose gel electrophoresis.

References in Supplemental Materials


39. Al-Mugotir, M.; Lovelace, J.J.; George, J.; Bessho, M.; Pal, D.; Struble, L.; Kolar, C.; Rana, S.; Natarajan, A.; Bessho, T.; et al. Selective Killing of Homologous Recombination-Deficient Cancer Cell Lines by Inhibitors of the RPA: RAD52 Protein-Protein Interaction. *PLoS One* **2021**, *16*, e0248941, doi:10.1371/journal.pone.0248941.

40. Huang, F.; Goyal, N.; Sullivan, K.; Hanamshet, K.; Patel, M.; Mazina, O.M.; Wang, C.X.; An, W.F.; Spoonamore, J.; Metkar, S.; et al. Targeting BRCA1- and BRCA2-Deficient Cells with RAD52 Small Molecule Inhibitors. *Nucleic Acids Res* **2016**, *44*, 4189–4199, doi:10.1093/nar/gkw087.

46. Zhu, S.; Peng, A. Non-Homologous End Joining Repair in Xenopus Egg Extract. *Sci Rep* **2016**, *6*, 27797, doi:10.1038/srep27797.

55. Ward, I.M.; Chen, J. Histone H2AX Is Phosphorylated in an ATR-Dependent Manner in Response to Replicational Stress. *J Biol Chem* **2001**, *276*, 47759–47762, doi:10.1074/jbc.C100569200.
